# Natural Bioactive Compounds Targeting FABP4 in Adipogenesis and Obesity: Evidence from In Vitro and In Vivo Studies

**DOI:** 10.3390/ijms27031306

**Published:** 2026-01-28

**Authors:** Jan Sobczyński, Filip Nowaczyński, Katarzyna Smolińska, Joanna Lachowicz-Radulska, Anna Serefko, Aleksandra Szopa

**Affiliations:** 1 Department of Clinical Pharmacy and Pharmaceutical Care, Medical University of Lublin, Chodźki St. 1, 20-093 Lublin, Poland; filip.nowczynski@umlub.edu.pl (F.N.); joanna.lachowicz-radulska@umlub.edu.pl (J.L.-R.); anna.serefko@umlub.edu.pl (A.S.); aleksandra.szopa@umlub.edu.pl (A.S.); 2 Doctoral School of the Medical University of Lublin, Chodźki St. 7, 20-093 Lublin, Poland; 3 Chronic Wounds Laboratory, Medical University of Lublin, Chodźki St. 7, 20-093 Lublin, Poland; katarzyna.smolinska@umlub.edu.pl

**Keywords:** FABP4, adipogenesis, obesity, natural products, phytochemicals, AMPK, PPARγ, inflammation

## Abstract

FABP4 (fatty acid-binding protein 4) is a lipid chaperone and secreted adipokine linking dysregulated fatty acid handling with inflammation, cellular stress, and insulin resistance in obesity. By modulating nuclear receptor signaling (notably PPARγ) and enhancing NF-κB/MAPK activation in adipocytes and macrophages, FABP4 contributes to maladaptive adipose remodeling and systemic metabolic decline. This review critically summarizes recent preclinical evidence on natural bioactive compounds that regulate FABP4 expression and associated adipogenic programs in models of adipogenesis and diet-induced obesity. Data from 3T3-L1/OP9 adipocytes, rodent studies, and selected alternative models indicate that many plant-derived extracts and phytochemicals (e.g., polyphenols, saponins, coumarins, terpenoids, and fermented products) down-regulate FABP4 at mRNA and/or protein levels. These effects are frequently accompanied by suppression of PPARγ/C/EBPα/SREBP1c signaling, activation of AMPK-related pathways, reduced lipid accumulation, and improved metabolic outcomes including lower weight gain, reduced adipocyte hypertrophy, improved steatosis, and favorable serum lipid profiles. Natural compounds from non-plant sources (animal- and microbe-derived metabolites) further broaden FABP4-targeting strategies, supporting FABP4 as a cross-class therapeutic node. Key translational barriers include poor extract standardization, incomplete identification of active constituents, limited oral bioavailability, microbiome-dependent variability, and scarce clinical validation. Future work should prioritize well-characterized lead scaffolds, targeted delivery, rational combinations, and standardized, adequately powered clinical trials assessing dose, durability of FABP4 suppression, and cardiometabolic safety.

## 1. Introduction

Obesity is regarded as one of the most pressing global health challenges, with a rapidly increasing prevalence across both developed and developing countries, affecting individuals of all age groups [1,2]. Beyond the issue of excessive fat accumulation, obesity is now recognized as a complex, chronic, relapsing disease characterized by profound metabolic, endocrine, and immunological disturbances. Its association with the development of type 2 diabetes, cardiovascular disease, metabolic dysfunction-associated fatty liver disease, certain cancers, and increased all-cause mortality is well-documented [1,2,3,4]. A hallmark of obesity is the persistent low-grade inflammation, also known as metaflammation, which stems from dysfunctional adipose tissue expansion and contributes to systemic insulin resistance and metabolic deterioration [5,6,7].

In obesity, adipose tissue undergoes structural and functional remodeling, marked by adipocyte hypertrophy, impaired adipogenesis, hypoxia, fibrosis, and increased infiltration of immune cells, particularly proinflammatory macrophages. These changes disrupt lipid storage capacity and promote ectopic lipid deposition in the liver, skeletal muscle, and pancreas, thereby exacerbating lipotoxicity and metabolic stress [8,9,10,11]. At the molecular level, obesity is characterized by dysregulated lipid handling, altered adipokine secretion, activation of inflammatory signaling pathways such as nuclear factor κB (NF-κB) and c-Jun N-terminal kinase (JNK), and impairment of insulin receptor signaling cascades [12]. Consequently, molecules that integrate lipid metabolism with inflammatory and stress signaling have gained attention as key drivers of obesity-associated complications.

Fatty acid-binding protein 4 (FABP4) contributes to the pathophysiology of obesity through its dual function as a lipid chaperone and signaling modulator. It regulates intracellular fatty acid trafficking and nuclear receptor stability, linking dysregulated lipid metabolism to transcriptional repression of adipogenic and insulin-sensitizing programs, as well as activation of stress and inflammatory pathways. By binding long-chain fatty acids, FABP4 delivers lipid ligands to nuclear receptors and transcriptional regulators, thereby modulating gene networks that control lipid storage and metabolic homeostasis [13,14]. FABP4 promotes the ubiquitination and proteasomal degradation of peroxisome proliferator-activated receptor γ (PPARγ), a process that suppresses adipogenic differentiation and insulin-sensitizing transcriptional activity [15]. Moreover, FABP4 is secreted from adipocytes through a non-classical, lipolysis-associated pathway and acts extracellularly as an adipokine that conveys metabolic signals to distant tissues [13,14].

Within adipocytes, FABP4 reprograms fatty acid handling toward proinflammatory and lipotoxic outcomes, contributing to maladaptive adipose tissue expansion and the development of visceral obesity. Elevated FABP4 expression in preadipocytes and mature adipocytes limits adipogenesis by down-regulating PPARγ, favoring hypertrophic rather than hyperplastic fat accumulation—a pattern linked to insulin resistance and metabolic dysfunction [15]. FABP4 levels are particularly high in visceral depots compared with subcutaneous fat and they are further elevated in obesity and diabetes, implicating depot-specific FABP4 expression in pathogenic adipose remodeling [14,15]. Secreted FABP4 augments adipose inflammation by promoting cytokine production and macrophage recruitment, intensifying hypoxia, fibrosis, and angiogenic dysfunction in obese adipose tissue [14,16].

In macrophages, FABP4 orchestrates lipid handling and inflammatory gene expression, converting metabolic cues into amplified cytokine production and foam cell formation that drive both adipose and vascular inflammation. FABP4 promotes cholesterol ester accumulation and foam cell generation typical of atherogenic processes, while its inhibition reduces inflammatory mediator release from activated macrophages [17]. Increased FABP4 activity enhances expression of proinflammatory cytokines such as tumor necrosis factor α (TNF-α) and interleukin-6 (IL-6), sustaining chronic adipose inflammation [17,18]. In macrophages, FABP4 also links fatty acid sensing to the activation of NF-κB and mitogen-activated protein kinase (MAPK) cascades, integrating metabolic stress with immune activation [13,18].

FABP4 acts as a central mediator connecting adipocyte lipid dysregulation and macrophage inflammation to whole-body insulin resistance. Through transcriptional repression of insulin-sensitizing genes and activation of inflammatory kinases, FABP4 disrupts insulin signaling across metabolic tissues. Its activity enhances obesity-associated JNK signaling that interferes with insulin receptor pathways in the liver and adipose tissue, whereas pharmacological inhibition of FABP4 improves insulin sensitivity [13,17]. Circulating FABP4 further acts hormonally to impair glucose metabolism in peripheral tissues, and elevated plasma FABP4 levels correlate with worsened metabolic and cardiovascular outcomes in humans [13,19]. In metabolic syndrome models, FABP4 inhibition restores insulin secretion and glucose tolerance while reducing islet inflammation and endoplasmic reticulum stress.

FABP4 serves as a bidirectional mediator of adipocyte–macrophage communication, establishing a feed-forward loop that amplifies local inflammation and propagates systemic metabolic deterioration. FABP4 released from adipocytes stimulates macrophage activation and cytokine expression, promoting adipose tissue infiltration and chronic inflammation in the adipose tissue [14,20]. Conversely, macrophage-derived FABP4 impairs insulin signaling and glucose uptake in adjacent adipocytes, directly influencing their metabolic responsiveness [20]. Evidence from cell-specific deletion studies and bone marrow chimeras demonstrates that cooperative FABP4 activity in both adipocytes and macrophages is required to reproduce the full inflammatory and metabolic phenotype of obesity, whereas its pharmacologic or genetic disruption reduces reciprocal activation and improves systemic metabolism [17,20].

FABP4 modulates multiple down-stream pathways that integrate lipid metabolism with inflammation and cellular stress responses. The PPARγ axis is the central one, in which FABP4-mediated proteasomal degradation of PPARγ decreases the expression of adipogenic and insulin-sensitizing target genes [15]. FABP4 activity also sustains JNK/MAPK and NF-κB signaling cascades, thereby promoting inflammatory transcription in both macrophages and adipose tissue [14,17,18]. Elevated FABP4 expression is associated with markers of endoplasmic reticulum stress such as glucose-regulated protein 78 (GRP78), activating transcription factor 6 (ATF6), and phosphorylated inositol-requiring enzyme 1α (IRE1α). Inhibition of FABP4 has been found to alleviate these stress responses [21]. In macrophages, FABP4 additionally modulates cholesterol trafficking and efflux, facilitating cholesterol ester accumulation and foam cell formation relevant to atherogenesis [17]. Collectively, these pathways define FABP4 as a molecular integrator of lipid signaling, inflammation, and stress responses that underpin obesity-related metabolic dysfunction.

**Figure 1:**
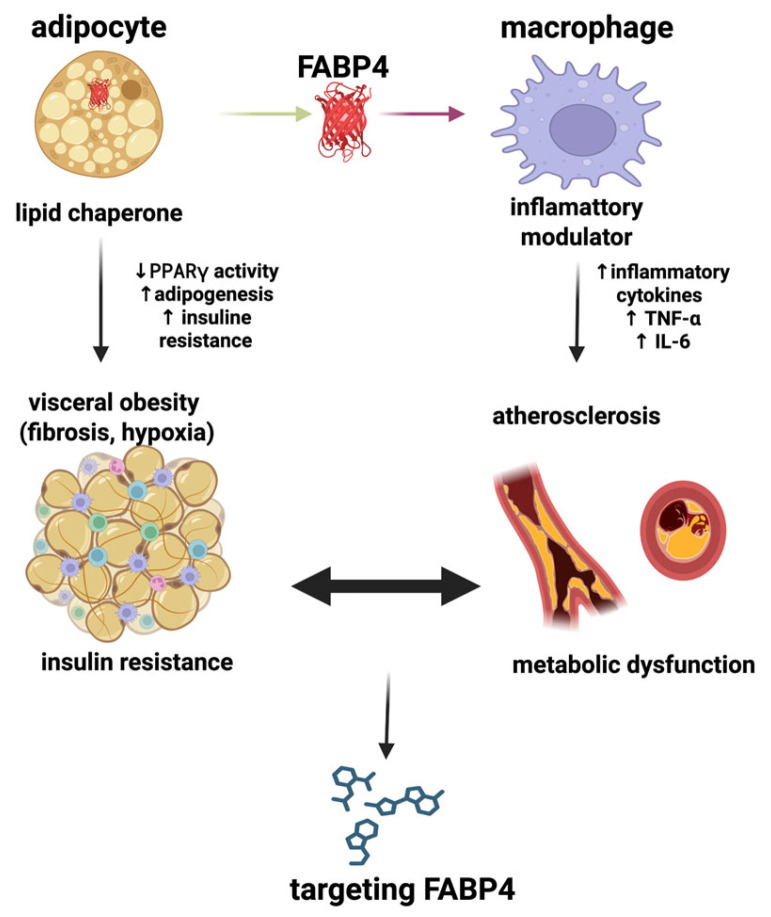
FABP4: a key player in obesity and metabolic dysfunction. Abbreviations: FABP4, fatty acid binding protein 4; IL-6, interleukin-6; PPARγ, peroxisome proliferator-activated receptor γ; and TNF-α, tumor necrosis factor α. Figure generated using BioRender.com.

To provide a concise graphical overview of key interconnections discussed above, the proposed role of FABP4 at the interface of adipose tissue dysfunction and inflammation was illustrated in Figure 1.

In parallel with the growing understanding of FABP4 biology, increasing attention has been given to bioactive natural compounds as potential modulators of FABP4-related pathways. A variety of plant-derived and other naturally sourced molecules have been shown in both in vitro adipocyte models and in vivo models of obesity to influence FABP4 expression, adipogenic transcriptional programs, lipid handling, and inflammatory signaling. These compounds may act through mechanisms such as modulation of the PPARγ axis, attenuation of NF-κB and MAPK activation, and improvement of metabolic stress responses, suggesting that FABP4 is not only a pathogenic mediator but also a pharmacologically responsive target.

Given the pivotal function of FABP4 in integrating lipid dysregulation, inflammatory signaling, and insulin resistance across adipocytes and macrophages, the targeting of FABP4 has emerged as a promising strategy for mitigating obesity-associated metabolic dysfunction. In this context, natural bioactive compounds have emerged as promising candidates for modulating FABP4 activity. The objective of this review is to systematically summarize and critically evaluate the current preclinical evidence on naturally derived compounds that regulate FABP4 expression and function in models of adipogenesis and obesity. The objective of this research is to identify natural compounds with documented effects on FABP4 modulation.

In order to accomplish this goal, our team conducted a review of original research regarding natural substances influencing the expression of FABP4, its mRNA, and adjacent factors in lipid metabolism pathways. Databases such as PubMed and Scopus were utilized to find such studies, using keywords “FABP4”, “FABP4 inhibitor”, “obesity”, “adipogenesis”, “natural compound”, and “plant-derived”, with preference to studies published in 2020–2025 period. We also decided to include both in vivo and in vitro studies, with preference to the former.

## 2. Plant Extracts/Substances Affecting FABP4

FABP4 has been identified as a promising therapeutic target due to its strong association with adipogenesis and lipid accumulation in cells. A series of recent studies has demonstrated the efficacy of various natural bioactive compounds in suppressing FABP4 expression and adipocyte differentiation in cellular and animal models. In this section, an attempt is made to provide a concise summary of recent observations on the activity of compounds of natural origin, based on in vitro and in vivo studies.

In recent years, several studies have been designed to investigate the regulatory effects of various natural compounds on FABP4 expression employing cellular and animal models of adipogenesis and obesity. The compounds described below have been grouped according to their chemical class or biological origin, while preserving the findings from each individual study.

### 2.1. Cereal- and Grain-Derived Compounds and Peptides

Natural products derived from cereal sources have been the subject of investigation with regard to their potential anti-adipogenic and FABP4-modulating activity.

In 2021, Oh et al. [22] utilized a network pharmacology to explore the anti-obesity potential of corn silk (*Stigma maydis*). The network pharmacology stands out as a robust method for elucidating multiple components [23], including signaling pathways, targets, and compounds present in significant metabolic pathways. The study identified several phytochemicals, including β-amyrone, ethyl linoleate, linoleic acid, oleic acid, and palmitic acid, as active substances capable of interacting with key adipogenic targets by assessing their interactions with significant molecular targets. In silico experiments demonstrated that β-amyrone exhibited the strongest molecular docking affinity for FABP4 (−13.2 kcal/mol), suggesting a direct inhibitory interaction [22]. Selected compounds were also predicted to influence the PPAR signaling pathway, through which FABP4 plays a central role in lipid transport and adipocyte differentiation [22]. Subsequently, Zhang et al. (2022) [24] provided in vivo confirmation of FABP4 modulation by corn peptide—a naturally occurring, biologically active peptide obtained through protease-catalyzed hydrolysis of corn. Moreover, studies using the C57BL/6J mouse model fed a high-fat diet (HFD) demonstrated that oral administration of corn peptide in combination with physical activity led to a substantial inhibition of FABP4 expression in murine adipose tissue [25]. The observed effect correlated with reduced body weight, adipocyte hypertrophy, and improved total cholesterol (TC) and low-density lipoprotein cholesterol (LDL-C) levels. These results suggest that corn derivatives might modulate adipogenesis by affecting FABP4 expression, providing a potential therapeutic tool for the prevention and/or treatment of obesity.

It is interesting to note that a significant down-regulation of both mRNA and protein levels of FABP4 was also observed following the administration of the polyphenol-rich extract of hulless barley (*Hordeum vulgare* L. var. nudum Hook. f.) [26]. It is noteworthy that the polyphenol extract of hulless barley exerted a range of additional biological effects, including a reduction in intracellular TC accumulation and lipid droplet formation, cell cycle arrest at the G0/G1 phase, and enhancement of antioxidant protection, as indicated by increased superoxide dismutase (SOD), catalase (CAT), and glutathione (GSH) activity. The up-regulation of a lipolytic enzyme—adipose triglyceride lipase—further supported the lipid-lowering activity of the above-mentioned extract. In the context of 3T3-L1 adipocytes, it was demonstrated that the treatment with the arginyl-fructose-enriched Jeju barley (*Hordeum vulgare* L.) extract (500 and 750 µg/mL) exerts a substantial reduction in lipid accumulation, concomitant with the suppression of adipocyte differentiation [27]. This effect was accompanied by a reduction in the expression of adipogenic transcription factors, including PPARγ and CCAAT-enhancer-binding proteins (C/EBPs) α, as well as their down-stream targets FABP4, fatty acid synthase (FAS), and sterol regulatory element-binding protein 1 (SREBP1) c. C57BL/6 mice were fed an HFD and given orally the barley extract for 8 weeks at a dose of 0.3 g/kg body weight. The treatment regimen resulted in (1) a significant reduction in body weight gain, (2) a decrease in visceral fat accumulation, (3) an improvement in plasma lipid profiles, i.e., a decrease in triglyceride (TG) and LDL-C, as well as an increase in high-density lipoprotein cholesterol (HDL-C), and (4) an increase in serum adiponectin levels.

### 2.2. Polyphenols and Phenolic Small Molecules

Polyphenolic compounds represent a substantial group of FABP4 modulators, exerting their primary effects through adipogenic transcriptional regulation and metabolic signaling pathways.

Genistin, a glycosylated form of the soy isoflavone genistein, has recently been identified as a potential agent that may produce an anti-adipogenic effect [28], possibly through the suppression of key adipogenic transcription factors. In the study by Choi et al. (2020) [29], genistin was found to down-regulate both mRNA and protein levels of FABP4 in 3T3-L1 adipocytes, and this effect was considered as a dose-dependent one. The reduction in FABP4 expression by genistin was accompanied by suppressing the expression of other adipogenic regulators such as PPARγ/EBPα, as well as by decreased expression of lipogenic enzymes including ATP citrate lyase (ACL), acetyl-CoA carboxylase (ACC) 1, and FAS. It is also important to note that genistin activated 5′-adenosine monophosphate-activated protein kinase (AMPK) α and reduced SREBP1c mRNA levels, suggesting [29] that modulation of the AMPK/SREBP1c pathway contributes to its anti-adipogenic mechanism. As previously observed with hulless barley extract [26], this effect was dose-dependent and accompanied by the suppression of the expression of other adipogenic regulators (e.g., PPARγ, C/EBPα, and fatty acid synthase, FAS).

In the study by Shih et al. (2023) [30], the anti-adipogenic effects of resveratrol butyrate esters were explored in 3T3-L1 adipocytes supplemented with MDI mixture consisting of 0.5 mM 3-isobutyl-methylxanthine, 1 mM dexamethasone, and 10 g/mL insulin. The study demonstrated that the novel derivatives of resveratrol significantly reduced intracellular lipid accumulation and TG content in a dose-dependent manner, outperforming native resveratrol. Noteworthy, resveratrol butyrate esters led to a down-regulation of the expression of FABP4 mRNA, and other adipogenic transcription factors, including PPARγ, C/EBPα, and FAS. The effect coincided with a significant increase in the phosphorylated 5′-adenosine monophosphate-activated protein kinase (p-AMPK)/AMPK ratio, indicating that FABP4 inhibition may be mediated through AMPK pathway activation, leading to a decreased adipocyte differentiation and lipid biosynthesis. The results of the study highlight the potential of resveratrol butyrate esters as promising modulators of FABP4 expression and as anti-obesity agents with enhanced bioavailability and efficacy as compared to native resveratrol [30].

Cinnamyl alcohol, a naturally occurring aromatic compound derived from cinnamon, has been demonstrated to exert anti-adipogenic effects by targeting early adipocyte differentiation [31,32]. In 3T3-L1 preadipocytes, cinnamyl alcohol treatment (6.25–25 μM) for 8 days significantly inhibited lipid accumulation and down-regulated the expression of key adipogenic markers, including FABP4, at both mRNA and protein levels in a concentration-dependent manner [33]. The study conducted by Choi and co-workers (2024) [33] demonstrated that cinnamyl alcohol exerts its inhibitory effect on adipogenic markers primarily during the early phase of adipocyte differentiation, particularly by blocking mitotic clonal expansion and arresting the cell cycle at the G0/G1 phase. Furthermore, cinnamyl alcohol modulated up-stream regulatory pathways by activating AMPKα and suppressing extracellular signal-regulated kinase (ERK) 1/2 phosphorylation [33]. These mechanisms have previously been shown to influence FABP4 expression.

In a recent study, isoeugenol, a phenylpropanoid compound found in clove and other aromatic plants [34,35], was identified as a potent inhibitor of adipogenesis in 3T3-L1 preadipocytes [36]. It was observed that isoeugenol significantly reduced both mRNA and protein levels of FABP4. This down-regulation was observed during the early phase of adipocyte differentiation, particularly during mitotic clonal expansion, a critical step for adipogenesis. Isoeugenol exerted its anti-adipogenic effect by arresting the cell cycle at the G0/G1 phase, reducing reactive oxygen species (ROS) levels, and attenuating phosphorylation of protein kinase B (AKT) and ERK-signaling pathways known to influence FABP4 expression [36]. The temporal aspect of the intervention was found to be pivotal, i.e., a two-day treatment with isoeugenol at the onset of differentiation resulted in a higher reduction in FABP4 and adipogenic markers when compared to the effect observed after the continuous eight-day exposure.

Salicortin, a bioactive phytochemical identified in *Salix pseudo-lasiogyne* twigs [37], was investigated using in vitro model by Kim and co-workers (2022) [38]. Salicortin exhibited a potent inhibitory effect on lipid accumulation, significantly reducing intracellular lipid content by up to 82% at a concentration of 50 μM. Salicortin was found to down-regulate the mRNA and protein levels of FABP4, as well as other key transcriptional regulators of adipogenesis, including PPARγ, C/EBPα, C/EBPβ, and the lipogenic enzyme FAS. The IC_50_ value for the anti-lipid accumulation effect of salicortin was reported to be 37.1 μM. Notably, these effects occurred without detectable toxicity, even at concentrations up to 200 μM [38].

### 2.3. Flavonoids and Biflavonoids

It is interesting to note that recent observations concerning the role of FABP4 in adipogenesis and lipid accumulation suggest that it could be a promising therapeutic target in obesity-associated metabolic disorders [39]. Cho and coworkers (2021) [40] studied the effect of oral administration of an extract derived from a shrub *Viburnum stellato-tomentosum*. The extract led to a significant reduction in FABP4 expression at both mRNA and protein levels in the white adipose tissue and liver of C57BL/6J mice with obesity induced by an HFD. The reduction was accompanied by decreased expression of other adipogenic markers such as lipogenesis-related C/EBPα and *Perilipin* (late marker of adipocyte differentiation). Furthermore, it led to suppression of adipocyte hypertrophy and hepatic steatosis. In addition, amentoflavone, a major bioactive compound identified in the *Viburnum stellato-tomentosum* extract, independently reduced FABP4 expression and improved insulin signaling in the skeletal muscle by up-regulating a signaling adapter protein insulin receptor substrate 1 (Irs1) and glucose transporter 1 (Glut1), a membrane protein that facilitates the transport of glucose and other hexoses across the cell membrane. These findings suggest that either the *Viburnum stellato-tomentosum* extract or amentoflavone exerts anti-obesity and glucose-lowering effects, at least in part, through the inhibition of FABP4-mediated lipid transport and adipocyte differentiation.

In studies by Hong et al. (2024) [41], Jee et al. (2024) [42], and Lee et al. (2020) [43], the anti-obesity effects of aqueous extracts from the king trumpet mushroom species *Pleurotus ferulae*, *Lycium chinense* Mill., and a perennial plant *Chrysanthemum morifolium*, respectively, were investigated. The findings demonstrated that these extracts exerted potent inhibitory effects on adipogenesis and lipid accumulation. In both 3T3-L1 preadipocyte cultures and HFD-induced obese mice, administration of the aqueous *Pleurotus ferulae* extract led to a significant reduction in body weight gain, fat mass, adipocyte hypertrophy, and serum lipid levels (TC and LDL-C) [41]. In a similar manner, oral administration of the aqueous extract of *Lycium chinense* resulted in a reduction in body weight gain, improvement in glucose tolerance, and amelioration of serum lipid profiles (a decrease in TC and TG). The treatment also attenuated hepatic steatosis and adipocyte hypertrophy [42]. At the molecular level, *Pleurotus ferulae* and *Lycium chinense* extracts suppressed the expression of key adipogenic and lipogenic regulators, including PPARγ, C/EBPα, and FABP4 [41,42]. Additionally, administration of the *Lycium chinense* extract decreased the expression of FAS and SREBP1c proteins. Real-time PCR analyses confirmed that the expression of FABP4 mRNA was significantly down-regulated during adipocyte differentiation upon treatment with the tested extracts, indicating a disruption of the fatty acid transport and storage pathways [42].

Finally, the *Chrysanthemum morifolium* flower extract suppressed FABP4 expression and activated the AMPK/sirtuin 1 (SIRT1) pathway, offering an alternative botanical approach to limit lipid accumulation in adipose tissue [43].

### 2.4. Coumarin Derivatives

3T3-L1 preadipocytes were used by Lamichhane et al. (2022) [44] to investigate anti-adipogenic effects of an extract and active molecules isolated from trifoliate orange (*Poncirus trifoliata*) fruits. Among the tested compounds, oxypeucedanin, a coumarin derivative obtained from the ethanolic extract, demonstrated the most potent inhibition of adipogenesis. The potential of coumarin compounds to inhibit the expression of FABP4 during the process of adipocyte differentiation was also investigated by Park and colleagues (2020) [45]. Scopolin, a coumarin glucoside derived from a boxthorn shrub *Lycium chinense*, significantly inhibited the expression of FABP4 during the process of adipocyte differentiation. The Oil Red O staining revealed a significant, dose-dependent reduction in lipid accumulation following treatment with oxypeucedanin [44] and scopolin [45]. Interestingly, both oxypeucedanin and scopolin led to a down-regulation of FABP4 at both the mRNA and protein levels, in addition to other adipogenic markers, including PPARγ, C/EBPα, SREBP1, and leptin [44,45]. Moreover, Park et al. (2020) [45] complemented in vitro observations with in vivo studies. The authors demonstrated that scopolin (20 and 40 mg/kg body weight/day) led to a reduced adipocyte size, hepatic steatosis, body weight gain, and serum insulin and leptin levels in ovariectomy-induced obese mice [45]. These findings suggest that the studied coumarin derivatives exert their anti-adipogenic and metabolic regulatory effects, at least in part, via down-regulation of FABP4 expression. Furthermore, these effects were accompanied by a reduction in lipid droplet formation, suggesting that oxypeucedanin caused a multi-level suppression of adipocyte differentiation.

### 2.5. Saponins and Triterpenoid Lactones

Saponins and associated triterpenoids have been observed to regulate FABP4 through AMPK-dependent mechanisms with consistent results.

The study by Oh and Chun (2022) [46] demonstrated the activity of saponins, highlighting the role of both native compounds and their metabolites. In their study, the authors used a treatment with ginsenoside compound K, a rare intestinal metabolite of ginseng saponins from *Panax ginseng* [47,48]. As a result, a significant reduction in lipid and TG accumulation and an arrest in cell cycle progression during mitotic clonal expansion were achieved. Bacoside-A, a dammarane-type triterpenoid saponin derived from the waterhyssop *Bacopa monnieri*, was found to possess a similar potential [49]. Interestingly, these compounds were able to down-regulate the expression of FABP4 at both the mRNA and protein levels in 3T3-L1 adipocytes in a dose-dependent manner (20–40 µM and 10–20 μg/mL for compound K and Bacoside-A, respectively) [46,49]. It was demonstrated that the suppression of FABP4 expression was mediated via the activation of the AMPK signaling pathway. Both saponin derivatives suppressed the expression of key transcription factors involved in adipogenesis, including PPARγ, C/EBPα, and SREBP1c. Another triterpenoid saponin, akebia saponin D (Asperosaponin VI), which was derived from the Japanese teasel (*Dipsacus asper*) [50], had anti-obesity activity [51]. The therapeutic effects observed in a murine model of metabolic syndrome induced by a HFD were partly mediated through modulation of the PPARγ/FABP4 signaling pathway. Akebia saponin D significantly improved metabolic parameters such as TG and fasting glucose levels, insulin resistance, and lipid accumulation in both murine adipose and intestinal tissues. At the molecular level, akebia saponin D treatment resulted in the down-regulation of FABP4 expression, both in vivo and in vitro, in intestinal epithelial cells challenged with linoleic acid. Furthermore, RNA sequencing and Western blotting analyses confirmed that akebia saponin D suppressed the expression of FABP4 alongside PPARγ, while enhancing the expression of several tight junction proteins, such as Zonula Occludens-1, Occludin, and Claudin-1, indicating improved integrity of the intestinal barrier [51]. These results position FABP4 as a key target of akebia saponin D in the mitigation of the intestinal barrier dysfunction and lipid-induced oxidative stress in metabolic syndrome. The findings suggest that saponins may be candidates for future metabolic therapeutics due to their FABP4 inhibition-mediated anti-obesity efficacy.

It is interesting to note that several steroid lactones, withanolides (including three newly identified ones: withasilolides G–I) isolated from *Withania somnifera* (ashwagandha) have demonstrated potent antia-dipogenic properties by interfering with key molecular pathways involved in adipocyte differentiation [52]. In 3T3-L1 preadipocyte models, all six withanolides tested by Lee et al. (2022) [52] significantly inhibited lipid droplet formation and reduced the expression of adipogenic markers, particularly FABP4 and adipsin, an adipokine, released by the fat tissue. Furthermore, they appeared to suppress FABP4 production in a dose-dependent manner at the protein level. It has also been suggested that withanolides enhanced lipid metabolism by up-regulating lipolytic genes of hormone-sensitive lipase and adipose triglyceride lipase while down-regulating SREBP1. Therefore, withanolides likely affect FABP4 at both transcriptional and translational levels, positioning ashwagandha as a promising natural source of future FABP4-targeted therapeutics preventing weight gain [52].

### 2.6. Marine-Derived Bioactive Compounds

Another group of natural compounds that have recently garnered attention regarding their anti-adipogenic properties is that of marine-derived sulfated polysaccharides. Filho and coworkers (2022) isolated a sulfated glucan from the green seaweed *Caulerpa sertularioides* (CS0.2 fraction) [53]. The CS0.2 fraction had the total sugar content of 63.6% ± 1.9%, sulfate content of 2.8% ± 0.4%, and the content of phenolic compounds < 0.1%. It was shown that the CS0.2 fraction significantly suppressed adipogenesis in 3T3-L1 cells. Among the key molecular effects, the expression of FABP4 mRNA was down-regulated by 5.4-fold following treatment with 50 µg/mL of CS0.2 fraction. It was accompanied by the decreased expression of other adipogenic and lipogenic regulators, including PPARγ, C/EBPα and C/EBPβ, SREBP1c, and a membrane glycoprotein CD36. Of particular interest is the observation that the CS0.2 fraction exhibited an anti-adipogenic activity at lower concentrations than typically required for comparable extracts; this effect was not associated with toxicity. Similarly, a monoterpene lactone (−)-loliolide and monogalactosyldiacylglycerols, isolated from a common brown seaweed—*Sargassum horneri* [54,55], inhibited lipid accumulation and decreased FABP4 protein levels, suggesting an anti-adipogenic action via reduced lipogenesis and enhanced lipolysis. Correspondingly, chlorophyll derivatives from a red seaweed *Grateloupia elliptica* suppressed the expression of FABP4 and other adipogenic proteins, thus underlining the potential of seaweed compounds in the prevention of obesity [56].

### 2.7. Terpenoid Compounds

Cedryl acetate, a naturally occurring sesquiterpene [57,58] that is used as aroma in perfumes and household products or as a flavoring agent in food (at levels not exceeding 1 mg/kg) [59], has been shown to possess the capacity to reduce the obesity induced by an HFD in a murine model [60]. Dietary supplementation of cedryl acetate at a dose of 100 mg/kg for 19 weeks resulted in a significant reduction in body weight gain, visceral fat accumulation, and hepatic steatosis. Additionally, the authors observed improvements in glucose tolerance and insulin sensitivity. The probable mechanisms by which cedryl acetate exerts these effects involved a significant suppression of the expression of key adipogenic and lipogenic genes in epididymal white adipose tissue, including FABP4, PPARγ, C/EBPα, and FAS. Furthermore, cedryl acetate enhanced the expression of thermogenic genes such as peroxisome proliferator-activated receptor γ coactivator 1α (PGC1α), positive regulatory domain containing 16 (PRDM16), and cell death activator CIDE-A [60]. These findings suggest a dual role for cedryl acetate in both inhibiting adipogenesis and promoting energy expenditure. However, as the concentration of cedryl acetate exceeded the currently acceptable intake levels, the study should be interpreted as an indication for further search for analogous molecules.

### 2.8. Whole Plant Extracts and Multi-Herbal Preparations

Complex botanical preparations also reduced FABP4 expression. In their study, Choi and colleagues (2021) [61] have demonstrated that FABP4 was significantly over-expressed in brown adipose tissue of mice (C57BL/6J) fed an HFD. Treatment with the aqueous tuber fleeceflower (*Polygonum multiflorum*) extract effectively reversed the elevation of FABP4 expression induced by HFD, restoring it to levels observed in mice fed a normal chow diet. This reduction was accompanied by the normalization of other lipolytic and thermogenic genes suggesting that the *Polygonum multiflorum* extract alleviates lipid overload and compensatory fatty acid oxidation in brown adipose tissue.

Another agent that demonstrated anti-adipogenic effects in 3T3-L1 preadipocytes was extracted from roots of east Asian hogweed (*Heracleum moellendorffii*), i.e., plant that has traditionally been used in east Asia for medicinal purposes [62,63]. In the study by Geum et al. (2021) [64], treatment with the *Heracleum moellendorffii* extract (100 and 200 µg/mL) led to a significant reduction in lipid accumulation, TC content, and the expression of key adipogenic and lipogenic proteins, including FABP4 in murine 3T3-L1 preadipocytes cells. In particular, the substantial down-regulation of FABP4 was manifested during both the early and late stages of adipogenesis. These observations indicate that the *Heracleum moellendorffii* extract exerts its regulatory effect on FABP4 expression as part of a comprehensive suppression of adipogenesis and lipid accumulation [64].

The anti-obesity and anti-adipogenic properties of two traditional herbal formulations—Gongmi tea (containing 11 herbs) and Gongmi So extract (an extract from 14 herbs)—were investigated [65] in both 3T3-L1 adipocytes and HFD-induced obese mice. The Gongmi tea at a dose of 300 μg/mL exerted the most potent activity, significantly reducing lipid accumulation in vitro, as confirmed by the Oil Red O staining. Furthermore, Western blot analysis demonstrated that Gongmi tea markedly suppressed the expression of FABP4, along with other key adipogenic transcription factors such as PPARγ and adiponectin. In addition, oral administration of Gongmi tea (200 mg/kg) to HFD-fed mice over a period of six weeks resulted in a significant reduction in murine body weight and epididymal adipose tissue mass, without any indication of kidney or liver toxicity. Collectively, these findings indicate that both Gongmi tea and Gongmi So extract might possess the potential to attenuate obesity by modulating FABP4-mediated adipogenesis [65], thereby making it a safe plant-derived therapeutic approach for metabolic syndrome.

A combination of the Japanese cornelian cherry *Cornus officinalis* and winter-berry currant *Ribes fasciculatum* extracts was tested in 3T3-L1 preadipocytes [66]. The concomitant administration of extracts at a dose of 10 μg/mL (ratio 7:3 of *Cornus officinalis* and *Ribes fasciculatum*, respectively) resulted in a significant reduction in FABP4 mRNA levels, along with other adipogenic markers. The mixture exhibited a more pronounced inhibitory effect on FABP4 compared to the individual extracts, suggesting a synergistic effect. The extract mixture administered at dose levels of 75 to 300 mg/kg over 12 weeks to HFD-induced obese mice resulted in a significant reduction in FABP4 expression in the adipose tissue, attenuation of hepatic steatosis, and in a decrease in body fat and weight [66]. These outcomes show that a concomitant application of herbal extracts may be more beneficial in respect to the inhibition of FABP4-mediated adipogenesis.

### 2.9. Fermented Natural Products

Apart from extracts derived from unmodified plant material, researchers’ interests include fermented natural products. Murine HFD model of obesity was used by Edward and co-workers (2023) [67] to investigate the effect of gochujang, a traditional Korean condiment, on obesity-related markers. Subsequently, a substantial down-regulation of FABP4 mRNA expression was observed in the livers of mice treated with traditional or commercial gochujang. Additionally, authors noted that gochujang consumption led to a reduction in murine body weight and improved insulin sensitivity. Accordingly, the potential of kombucha fermented from oolong and yellow tea (*Camellia sinensis*) in supporting weight management was investigated [68], with a particular interest in its impact on digestive enzymes and adipocyte differentiation. In OP9 adipocyte models, both oolong and yellow tea kombucha significantly down-regulated FABP4 gene and protein expression, as well as key adipogenic regulators including PPARγ, C/EBPα, and SREBP1c. RT-qPCR and Western blot analyses confirmed a dose-dependent decrease in FABP4 expression, which appeared to correlate with the reduced accumulation of lipid droplet and TG content in adipocytes. Further observations made in in vivo models validated these suggestions. In a recent study, Tran et al. (2024) [69] have found that kombucha derived from *Camellia sinensis* leaf exhibited anti-obesity effects in *Drosophila* models through the augmentation of lipase activity, thereby facilitating lipolysis. It is possible that the increased content of polyphenolic and organic acid produced during fermentation contributed to these effects. Thus, kombucha may have a dual anti-obesity mechanism—via the inhibition of digestive enzymes and the suppression of adipogenesis through FABP4 modulation. Moreover, Liu and co-workers (2024) [68] noted that kombucha regulated glucose metabolism. This finding highlights its potential as a plant-based functional beverage for the dietary management of obesity and metabolic dysfunction.

### 2.10. Novel Delivery Approaches

In a novel study, Li et al. (2024) [70] demonstrated the potential of photodynamic therapy with exosome-like nanovesicles (HPExos) loaded with the St. John’s wort (*Hypericum perforatum*) extract used as a photosensitizer in the treatment of obesity. Following administration in HFD-induced obese mice, HPExos were found to accumulate selectively in visceral adipose tissue, where light activation triggered an increased ROS production, promoting apoptosis in mature adipocytes. Importantly, the treatment in both in vivo (3T3-L1 adipocytes) and in vivo (HFD-fed mice) models yielded significantly down-regulated key adipogenic and lipogenic markers, including FABP4, PPARγ, C/EBPα, and SREBP. It is considered that this molecular effect was associated with reduced lipid accumulation, improved insulin sensitivity, and normalization of blood lipid profiles. These findings suggest that the nanovesicular St. John’s wort extract could be a novel, plant-derived agent capable of selectively targeting the adipose tissue and suppressing FABP4, offering a biocompatible, natural strategy for managing obesity and metabolic dysregulation.

### 2.11. Structural Diversity of Plant-Based Compounds Influencing FABP4

FABP4 is characterized by a 10-stranded β-barrel forming a hydrophobic cavity, capped by a helix-turn-helix portal region enriched in polar residues such as Arg106 and Tyr128 [71]. This architecture enables FABP4 to accommodate amphiphilic ligands, typically long-chain fatty acids, through deep insertion of the hydrophobic tail and anchoring of the polar headgroup at the portal. Plant-derived compounds exhibit diverse structural motifs that influence their potential to engage FABP4 either through canonical deep-pocket binding or alternative shallow/portal interactions [72].

Andrographolide, a diterpenoid lactone from green chiretta *Andrographis paniculata*, has emerged as a direct FABP4 inhibitor [73]. Fluorescence displacement assays demonstrated a competitive binding of andrographolide to FABP4, suggesting occupation of the ligand pocket and interference with fatty acid transport. In monosodium iodoacetate osteoarthritis-induced rat model, andrographolide reduced FABP4-driven fatty acid oxidation and reactive oxygen species, leading to decreased NF-κB activation and cartilage degradation. These findings position andrographolide as a promising natural scaffold for FABP4-targeted therapies, combining biochemical evidence with functional outcomes in vivo [73].

Ursolic acid, a pentacyclic triterpenoid abundant in many medicinal plants, influences FABP4 indirectly through modulation of the FABP4–PPARγ axis [74]. In triple-negative breast cancer cells, ursolic acid enhanced PPARγ activity and altered FABP4 expression, with knockdown experiments confirming a role of FABP4 in mediating anti-proliferative effects of this compound. While structural binding to FABP4 remains unproven, these pathway-level interactions highlight ursolic acid potential to regulate FABP4-dependent signaling [74]. Future biophysical assays and crystallography studies are expected to clarify whether ursolic acid engages FABP4 directly or acts up-stream via transcriptional control.

Rosmarinic acid, a polyphenolic compound found, for example, in rosemary and basil, has been linked to FABP4 regulation through PPAR signaling [75]. In lung adenocarcinoma models, the treatment with rosmarinic acid up-regulated FABP4 and PPARγ expression, activating apoptotic pathways and reducing tumor proliferation. It suggests a functional role of FABP4 in mediating the anticancer effects of rosmarinic acid, although direct ligand binding has not been demonstrated [75]. These findings underscore the importance of distinguishing between the transcriptional modulation and physical interaction when evaluating FABP4-targeted phytochemicals.

Oleanolic acid, another pentacyclic triterpenoid, and its derivatives have shown activity in metabolic regulation via FABP4-related pathways [76]. The derivative HA-20 suppressed adipogenesis in 3T3-L1 cells and HFD-fed mice by down-regulating PPARγ and FABP4 expression, reducing lipid accumulation and body weight. Although these effects indicate an involvement of the FABP4 pathway [76], an evidence of direct binding is lacking. Structural studies are needed to determine whether oleanolic acid interacts with FABP4 at the portal region or whether it acts primarily through transcriptional mechanisms.

Linoleic acid, a polyunsaturated fatty acid abundant in plant oils, represents the canonical FABP4 ligand [77]. Crystallographic data (PDB 2Q9S) confirm deep insertion of its hydrocarbon chain into the FABP4 β-barrel and anchoring of the carboxylate at the portal, establishing a benchmark for ligand engagement. This validated binding mode provides a structural reference for assessing other plant-derived compounds, particularly those with amphiphilic profiles [77]. The role of linoleic acid shows how natural fatty acids directly influence FABP4 function in lipid transport and metabolic regulation.

To facilitate a comparative overview of the available evidence, Table 1 summarizes plant-derived natural compounds and related preparations reported to modulate FABP4 expression in the context of adipogenesis and obesity. The table integrates data from in vitro and in vivo studies, highlighting the source material, identified active substances or substance groups, reported biological effects, as well as key advantages and limitations of each study.

It is interesting to note that the results of the above-mentioned studies appear to show a consistent pattern. It seems that diverse plant-derived compounds may modulate adipogenic pathways through the inhibition of FABP4 expression. These findings tentatively support the hypothesis that FABP4 functions as a convergence point for the anti-obesity effects of multiple phytochemicals. By suppressing FABP4 activity during the early and late stages of adipocyte differentiation, these agents could be a promising option in the dietary or pharmacological management of obesity and related metabolic disorders.

**Figure 2:**
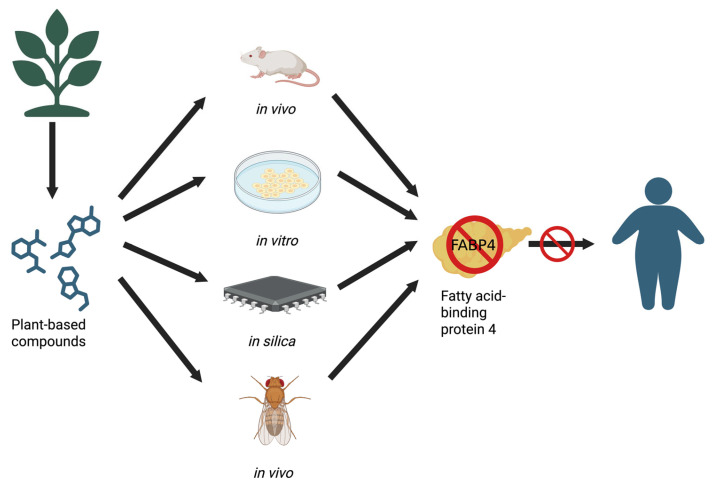
Overview of research approaches used to identify plant-based modulators of FABP4 in obesity-related studies. Figure generated using BioRender.com.

A schematic overview of the models and strategies applied in studies investigating plant-based FABP4 inhibition is presented in Figure 2.

## 3. Animal- and Microbe-Derived Bioactive Compounds Modulating FABP4 Expression in Adipogenesis

As it turns out, biologically active molecules possessing potential to suppress adipogenesis through down-regulation of FABP4 expression can be obtained from animal tissues and bacteria. In an in vivo study, protein hydrolysates derived from *Protaetia brevitarsis* larvae reduced obesity and hepatic inflammation in the HFD-fed mice [78]. A significant inhibition of FABP4 activity in the liver and adipose tissue was observed, alongside with the reduced levels of key markers, like leptin, PPARγ, and AMPK-α2. Further, Lee et al. (2022) [79] demonstrated that cell-free metabolites (CMRO6) from *Bacillus ginsengihumi* affect the expression of certain genes associated with adipogenesis, such as FABP4, along with other relevant genes like PPARγ, C/EBPα, SREBP1c, FAS, and ACC in 3T3-L1 adipocytes. It is interesting to note that the CMRO6 treatment led to a dose-dependent reduction in lipid accumulation, as visualized by the Oil Red O staining, and decreased FABP4 protein expression, as confirmed by Western blot [79]. These outcomes suggest that CMRO6 modulates lipid metabolism and adipocyte differentiation by targeting both up-stream transcription factors and FABP4, positioning this probiotic as a promising anti-obesity agent that could yield metabolic improvement. Likewise, Oh et al. (2021) [80] investigated the anti-adipogenic and insulin-sensitizing effects of cell-free extracts from three newly identified strains of *Lactobacillus plantarum*, i.e., LP, LS, and L14, using 3T3-L1 adipocytes. All three extracts significantly reduced lipid accumulation and suppressed the expression of key adipogenic markers, including PPARγ, C/EBPα, and notably, FABP4. The L14 strain extract demonstrated the most potent inhibitory effect on both the mRNA and protein expression levels of FABP4, exhibiting a dose-dependent response. Moreover, extracts from LS as well as L14 strains stimulated glucose uptake in maturing adipocytes, whereas the L14 extract reduced TNF-α levels, suggesting improved insulin sensitivity [80].

In their recent studies, Mahdavi and collaborators (2025) [81] evaluated the in vivo effects of gassericin A, a bacteriocin derived from *Lactobacillus gasseri* [82], on obesity and metabolic biomarkers in mice fed with a high-fat and high-sugar diet. While gassericin A promoted weight gain and abdominal fat accumulation, it also exerted a protective metabolic effect, including improved fasting blood glucose, liver function, lipid profile, and antioxidant status. Notably, the expression of the FABP4 (422/ap2) gene was significantly increased following treatment with gassericin A, suggesting enhanced adipogenesis and lipid handling in mesenteric adipose tissue. This up-regulation of FABP4 coincided with increased adipocyte hyperplasia but reduced adipocyte size [81], a pattern that is associated with metabolically healthier fat expansion. The preceding in vitro study [83] had revealed that gassericin A exerted no significant impact on the morphology or viability of the 3T3-L1 cell line. However, an important observation was that the cell number increased substantially, reaching almost double the level achieved in the control groups. Consequently, these findings [81,83] imply that although gassericin A promotes fat accumulation, it could concomitantly modulate FABP4 to favor hyperplastic over hypertrophic adipose remodeling, thereby attenuating obesity-related complications. Further research is warranted to determine the long-term effects of FABP4 up-regulation under such conditions.

To complement the overview of plant-derived compounds, Table 2 summarizes non-plant natural sources, including animal- and microbe-derived bioactive substances, reported to modulate FABP4 expression in preclinical models of adipogenesis and obesity. The table highlights the source organisms, identified bioactive compounds or metabolite fractions, observed biological effects, and key strengths and limitations of the available in vitro and in vivo studies, thereby providing insight into the broader landscape of FABP4 regulation beyond plant-based interventions.

## 4. Advantages and Limitations of Natural-Derived Compounds Targeting FABP4

Plant-derived bioactive compounds represent a diverse and chemically rich source of potential FABP4 modulators, offering several advantages over conventional synthetic agents. It has been demonstrated that a significant number of phytochemicals manifest pleiotropic biological activities, thereby enabling the simultaneous modulation of FABP4 expression and up-stream adipogenic regulators, including PPARγ, C/EBPα, SREBP1c, and AMPK-related pathways [84,85]. It is hypothesized that this multi-target mode of action may be particularly advantageous in complex metabolic disorders, such as obesity, where adipogenesis, lipid transport, inflammation, and insulin resistance are closely interconnected [86,87,88]. Moreover, there is a prevalent perception that plant-derived compounds are generally considered as safer and better tolerated by patients. A long-term dietary exposure or traditional use support this notion. It may facilitate the development of plant-derived products as nutraceuticals or functional food components [89,90].

Notwithstanding the aforementioned advantages, significant limitations concerning the translational process of results obtained in pre-clinical studies to human population have been noted. A significant challenge is posed by the lack of standardized extracts. It is due to the variability in phytochemical composition, which is a consequence of differences in plant genotype, cultivation conditions, harvesting time, and extraction procedures [91] (pp. 144–161). This heterogeneity impedes reproducibility across studies and limits meaningful comparison of biological effects. Furthermore, sometimes it is difficult to identify the active substance responsible for FABP4 modulation, which makes it impossible to carry out an analysis of the structure–activity relationship and to perform a rational optimization of plant-derived anti-obesity agents.

From a regulatory perspective, additional challenges arise from the use of complex plant extracts. The selection of extraction solvent (e.g., ethanol, methanol, acetone, or other organic solvents) has the potential to influence both the phytochemical profile and the safety of the final preparation, particularly in cases where residual solvents remain. Regulatory authorities mandate stringent oversight of solvent residues, batch-to-batch consistency, and comprehensive characterization of extract composition, a practice that is frequently absent in preclinical studies. Furthermore, a paucity of comprehensive clinical safety data exists for a considerable number of plant-derived extracts, particularly with regard to long-term use, interactions with conventional medications, and utilization in vulnerable populations [92]. These discrepancies have the potential to impede the regulatory approval process and constrain the translation of promising preclinical findings into clinically viable products.

A further critical limitation is poor bioavailability, which concerns a considerable number of polyphenols, flavonoids, and terpenoids. It is due to the following three factors: low solubility, rapid metabolism, and limited intestinal absorption [93,94,95]. Consequently, it may be difficult to reach concentrations of active compounds required to suppress FABP4 expression in humans. Additionally, the process of metabolism by gut microbiota has been demonstrated to exert a substantial influence on the biological activity of phytochemicals, thereby contributing to interindividual variability in metabolic responses [96]. Notwithstanding the widespread acceptance of their safety, there is a possibility that certain plant-derived compounds may exert off-target effects, induce endocrine interference, or demonstrate dose-dependent toxicity, particularly following chronic or high-dose exposure. This underscores the necessity for rigorous safety evaluation [97,98].

Finally, most available data discussed in this review are derived from preclinical models, predominantly 3T3-L1 adipocytes and HFD-induced obesity models in rodents. Various studies produced different outcomes, especially in regard to in vivo experiments. Whilst these systems are valuable for mechanistic insights, they do not fully reproduce human metabolic complexity. Studies in different animal models are needed to assess the possible metabolism of new bioactive compounds and to plan clinical trials. Moreover, the pharmacokinetics or effects of various dosing regimens of these natural agents have not been thoroughly studied. The lack of clinical trials limits conclusions regarding efficacy, optimal dosing, and long-term safety in humans [99].

## 5. Conclusions and Future Perspectives on Naturally Derived FABP4 Inhibitors

Preclinical evidence supports the hypothesis that FABP4 is a promising molecular target for the modulation of adipogenesis and lipid metabolism by plant-derived bioactive compounds. A considerable number of phytochemicals and botanical preparations have been shown to reduce FABP4 expression, inhibit adipocyte differentiation, and enhance metabolic parameters in both cellular and animal models of obesity; the obtained results underscore the therapeutic significance of interventions that target FABP4.

Future research should prioritize the identification of the most promising lead structures, particularly compounds that consistently suppress FABP4 expression at both the mRNA and protein levels while exhibiting favorable pharmacokinetic properties. Polyphenols, saponins, coumarin derivatives, and selected terpenoids are currently considered as the most promising compounds. However, further structure–activity relationship studies are required to enhance potency, selectivity, and metabolic stability of lead candidates.

To surmount bioavailability problems, there is a necessity to apply advanced delivery strategies, including nanoformulations, manipulation of amorphous state, encapsulation techniques, and targeted delivery systems to adipose tissue. Such approaches have the potential to enhance tissue specificity, reduce systemic exposure, and mitigate potential adverse effects. Furthermore, synergistic strategies, combining plant-derived FABP4 inhibitors with other metabolic modulators such as AMPK activators, anti-inflammatory agents, or microbiota-targeting interventions, may potentiate the treatment efficacy through coordinated multi-pathway regulation.

Importantly, future studies should also address the long-term safety and physiological consequences of sustained FABP4 modulation, given the central role of FABP4 in lipid trafficking and energy homeostasis. To achieve translational progress and to establish the efficacy and safety of plant-derived FABP4 inhibitors in humans, standardized extract formulations, robust in vivo validation, and ultimately well-controlled clinical trials are needed.

## Figures and Tables

**Table 1:** Summary of plant-derived natural compounds modulating FABP4 expression in adipogenesis and obesity.

Plant or Material	Substance or Substance Group, if Described	Effect	Advantages and Limitations of the Study	Ref.
**Polyphenols and phenolic compounds**				
*Glycine max*	Isoflavones, genistin	Suppressed adipocyte differentiation; lower FABP4, PPARγ, and C/EBPα expression	In vitro study	[29]
N/A	Resveratrol and its butyrate esters	Lower expression of FABP4 mRNA; the study suggested that AMP-activated protein kinase is involved in lipid metabolism	In vitro study	[30]
N/A	Isoeugenol	Lower lipid accumulation in cell culture; lower expression of FABP4, PPARγ, and C/EBPα factors	In vitro study	[36]
N/A	Cinnamyl alcohol	Inhibited lipid accumulation; down-regulated expression of FABP4, PPARγ, C/EBPα, FAS, and SREBP1c	This study described the mechanism through which cinnamyl alcohol suppresses adipogenesis	[33]
*Salix pseudo-lasiogyne*	Salicortin	Lower levels of FAS, C/EBPα, PPARγ, C/EBPβ, and FABP4 expression	In vitro study	[38]
N/A	Ursolic acid	Lower FABP4 and PPARγ mRNA levels in tumor tissues	In vitro study	[74]
*Camellia sinensis*	N/A	Lower inflammatory reaction and lipase activity	An in vivo study corresponding to Liu et al. study	[69]
*Hypericum perforatum*	This study utilized exosome-like nanovesicles	PPARγ, C/EBPα, SREBP, and FABP4 expression inhibition both in vitro and in vivo	In vitro and in vivo study	[70]
N/A	Rosmarinic acid	Down-regulation of FABP4 and PPARγ expression	In vitro and in vivo study	[75]
**Flavonoids and biflavonoids**				
*Viburnum stellato-tomentosum*	Amentoflavone	Reduced expression of lipogenesis-related genes	In vivo data but the study did not describe the effect of the tested extract on inflammation	[40]
*Lycium chinense*	Scopolin	Lowered expression of FABP4 mRNA; lowered adipocyte differentiation	In vitro study	[45]
**Saponins and triterpenoids**				
*Panax ginseng*	Ginsenoside compound K	Inhibited expression of adipogenic marker genes, including FABP4; the study suggests that active compound inhibits adipocyte differentiation	In vitro study	[46]
*Bacopa monniera*	Bacoside-A	Down-regulation of FABP4 mRNA expression	In vitro study	[49]
N/A	Asperosaponin VI (akebia saponin D)	Reduced FABP4 and PPARγ expression	In vivo study; the study also considered the effect on microbiota	[51]
*Withania somnifera*	Withanolides	Lower FABP4 and adipsin expression	In vitro study	[52]
N/A	Oleanolic acid and HA-20 derivative	Lowered PPARγ, C/EBPα, and FABP4 expression	In vitro and in vivo study	[76]
**Coumarins**				
*Poncirus trifoliata*	Oxypeucedanin; other coumarin derivatives	Reduced expression of SREBP1, PPARγ, and FABP4	In vitro study	[44]
**Polysaccharides and peptide derivatives**				
*Zea mays*, specifically *Stigma maydis*	β-amyrone	Postulated direct inhibitory interaction; high affinity to target proteins	In silica study—no in vitro or in vivo data; no information on the expression of target gene	[22]
*Zea mays*	Corn peptide	Reduced obesity in mice	In vivo confirmation of Oh et al. study	[24]
*Pleurotus ferulae*	Polysaccharides	Reduced body mass gain in a murine model; lower FABP4 expression in a cellular model	In vitro and in vivo studies	[41]
*Hordeum vulgare*	Enriched barley extract	Significant reduction in C/EBPα, PPARγ, FAS, and FABP4 expression; inhibition of adipocyte differentiation	In vivo study	[27]
**Cereal polyphenol extracts**				
*Hordeum vulgare* var. nudum	N/A	Inhibition of adipogenic regulators—PPARγ, C/EBPα, FAS, and FABP4; postulated regulation of mRNA and proteins of adipogenic genes	In vitro study	[26]
**Marine-derived compounds**				
*Caulerpa sertularioides*	Sulfated glucan (CS0.2 fraction)	Lower expression of FABP4 mRNA and other adipogenic factors, such asC/EBPα, C/EBPβ, and PPARγ	In vitro study	[53]
*Sargassum horneri*	Mono-galactosyl-diacyl-glyceroles	Reduced levels of triglyceroles and free fatty acids	In vitro study	[54]
*Sargassum horneri*	(–)-loliolide	Inhibition of lipid metabolism—lowered FABP4 expression, postulated regulations of lipolysis	In vitro study	[55]
*Grateloupia elliptica*	Chlorophyll derivatives	Suppressed expression of SREBP1, PPARγ, C/EBPα, and FABP4	In vitro study	[56]
**Whole plant extracts and mixtures**				
*Polygonum multiflorum*	N/A	Reduced FABP4-coding gene expression; administration of *P. multiflorum* extract led to the reversal of the high-fat-induced lipid metabolism	In vivo study in mice	[61]
*Heracleum moellendorffii*	N/A	Blocked expression of lipid-accumulated proteins in adipocytes	In vitro study; the active substance has not been specified	[64]
*Chrysanthemum morifolium* flower	N/A	Suppressed FABP4 expression and activation of the AMPK/SIRT1 pathway	In vitro study; the active substance has not been specified	[43]
Gongmi tea (11 plants extract) Gongmi so (14 plants extract)	N/A	Inhibition of FABP4, PPARγ, and adiponectin; the extracts did not exhibit toxicity in mice	In vitro and in vivo studies	[65]
*Cornus officinalis, Ribes fasciculatum*	N/A	Lowered PPARγ, C/EBPα, SREBP1, and FABP4 expression in white fat tissue	In vivo study; the active substance has not been specified	[66]
*Lycium chinense*	N/A	Lower FABP4, FAS, and SREBP1c expression; lower food intake	In vitro and in vivo studies	[42]
**Fermented natural products**				
Gochujang	N/A	Down-regulation of FABP4 mRNA; lower murine body weight, improved insulin sensitivity	In vivo study	[67]
Kombucha from *Camellia sinensis*	Polyphenoles	Lowered expression of FABP4, PPARγ, C/EBPα, and SREBP1c	In vitro study	[68]
**Other compounds**				
N/A	Cedryl acetate	Lower insulin sensitivity, lower food intake, and reduced adipocyte size in adipose tissue; suppressed FAB4 expression	In vivo study	[60]
*Andrographis paniculata*	Andrographolide	Direct FABP4 inhibitor	In vitro and in vivo study	[73]
N/A	Linoleic acid	Natural FABP4 ligand	Crystallographical in vitro study	[77]

AMPK, 5′-adenosine monophosphate-activated protein kinase; C/EBP, CCAAT-enhancer binding protein; FABP4, fatty acid-binding protein 4; FAS, fatty acid synthase; N/A, not applicable; PPARγ, peroxisome proliferator-activated receptor γ; SIRT1, sirtuin 1; and SREBP1, sterol regulatory element-binding protein 1.

**Table 2:** Non-plant-derived bioactive compounds modulating FABP4 expression in preclinical models of adipogenesis and obesity.

Non-Plant Source	Substance or Substance Group, if Described	Effect	Advantages and Limitations of the Study	Ref.
*Protaetia brevitarsis*	Protein hydrolysates	Inhibition of FABP4 synthesis in liver and adipose tissue	In vivo study	[78]
*Bacillus ginsengihumi*	CMRO6 cell-free metabolites	Down-regulated expression of C/EBPβ, C/EBPα, PPARγ, SREBP1c, FAS, and FABP4	In vitro study	[79]
*Lactobacillus plantarum*	Three cell-free extracts	All three extracts exhibited varying levels of adipogenic markers expression, FABP4 included; lowered TNF-α levels	In vitro study	[80]
*Lactobacillus gasseri*	Gassericin A	Higher expression of FABP4 coupled with expansion of metabolically healthier fatty tissue; the treatment caused an increase in adipocyte count	In vitro study	[83]
*Lactobacillus gasseri*	Gassericin A	The results were analogical to the study by Taghizad et al. [83]	In vivo study; confirmation of results obtained in the study by Taghizad et al. [83]	[81]

C/EBP, CCAAT-enhancer binding protein; FABP4, fatty acid-binding protein 4; FAS, fatty acid synthase; PPARγ, peroxisome proliferator-activated receptor γ; SREBP1, sterol regulatory element-binding protein 1; and TNF-α, tumor necrosis factor α.

## Data Availability

No new data were created or analyzed in this study. Data sharing is not applicable to this article.

## Abbreviations

The following abbreviations are used in this manuscript:

ACC	Acetyl-CoA carboxylase
ACL	ATP citrate lyase
AKT	Protein kinase B
AMPK	5′-adenosine monophosphate-activated protein kinase
ATF6	Activating transcription factor 6
C/EBP	CCAAT-enhancer binding protein
CAT	Catalase
ERK	Extracellular signal-regulated kinase
FABP4	Fatty acid-binding protein 4
FAS	Fatty acid synthase
Glut1	Glucose transporter 1
GRP78	Glucose-regulated protein 78
GSH	Glutathione
HDL-C	High-density lipoprotein cholesterol
HFD	High-fat diet
HPExos	Exosome-like nanovesicles
IL-6	Interleukin-6
IRE1α	Phosphorylated inositol-requiring enzyme 1α
Irs1	Insulin receptor substrate 1
JNK	c-Jun N-terminal kinase
LDL-C	Low-density lipoprotein cholesterol
MAPK	Mitogen-activated protein kinase
NF-κB	Nuclear factor κB
p-AMPK	Phosphorylated 5′-adenosine monophosphate-activated protein kinase
PGC1α	Peroxisome proliferator-activated receptor γ coactivator 1α
PPARγ	Peroxisome proliferator-activated receptor γ
PRDM16	Positive regulatory domain containing 16
ROS	Reactive oxygen species
SIRT1	Sirtuin 1
SOD	Superoxide dismutase
SREBP1	Sterol regulatory element-binding protein 1
TC	Total cholesterol
TG	Triglyceride
TNF-α	Tumor necrosis factor α
